# Supplementary material for: Dynamics of CO photooxidation to CO2 on rutile (110)
Source: Commun Chem. 2026 Mar 10;9:127. doi: 10.1038/s42004-026-01901-2 (PMC13009378; doi:10.1038/s42004-026-01901-2)
Supplement: Supplementary file 2 — Supporting Information: Dynamics of CO Photooxidation to CO2 on Rutile (110) [file 42004_2026_1901_MOESM2_ESM.pdf]

Supporting Information: Dynamics of CO Photooxidation to CO_2 on Rutile (110)

Helena Gleißner 1,3,4; Michael Wagstaffe 1,10; Lukas Wenthaus 2; Adrian Domínguez-Castro 5; Verena Gupta 5; Simon Chung 1; Steffen Palutke 2,11; Siarhei Dziarzhytski 2; Dmytro Kutnyakhov 2; Michael Heber 2,11; Günter Brenner 2; Harald Redlin 2; Federico Pressacco 2; Adriel Domínguez Garcia 5,6,7; Thomas Frauenheim 8,9; Heshmat Noei 1,3*; Andreas Stierle 1,3,4*.

1 Centre for X-ray and Nanoscience CXNS, Deutsches Elektronen-Synchrotron DESY, Hamburg 22603, Germany

2 Deutsches Elektronen-Synchrotron DESY, Hamburg 22603, Germany

3 The Hamburg Centre for Ultrafast Imaging, Hamburg 22761, Germany

4 Fachbereich Physik, Universität Hamburg, Hamburg 20355, Germany

5 Bremen Center for Computational Materials Science, Universität Bremen, Bremen 28359, Germany

6 Computational Science Research Center (CSRC) Beijing 100193, China

7 Computational Science Applied Research (CSAR) Institute Shenzhen, Shenzhen 518110, China

8 School of Science, Constructor University, Bremen 28759, Germany

9 Institute for Advanced Study, Changdu University, Chengdu 610106, China

10 current: Fraunhofer Institute for Solid State Physics IAF, 79108 Freiburg,Germany

11 current: European XFEL, Schenefeld 22869, Germany

* Corresponding Authors:

Heshmat Noei heshmat.noei@desy.de;

Andreas Stierle andreas.stierle@desy.de

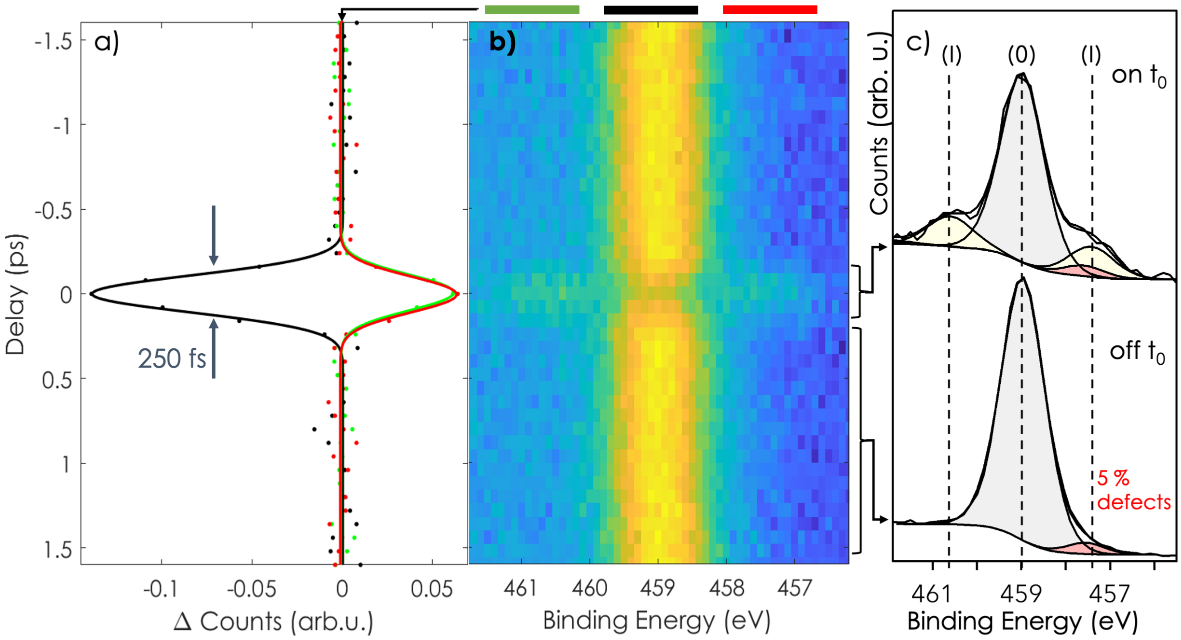

Fig. S1: Intensity profile (a) of the lattice peak and corresponding sidebands taken from distinct energy regions of pßthe XP map (b) of the Ti 2p core level. The green (459.9-461.3 eV), black (458.3-459.7 eV), and red (456.7-458.1 eV) bars indicate the binned energy region for the line profile. c) XP spectra during (top) and off time-zero (bottom).

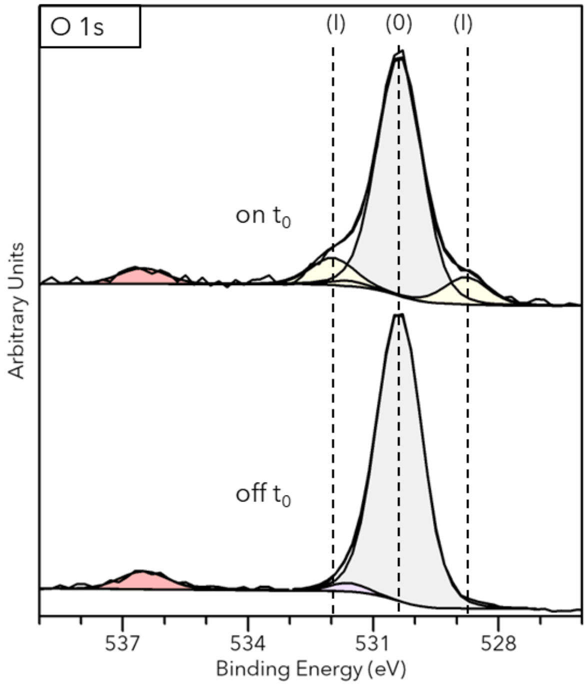

Fig. S2: Formation of first-order sidebands (I) in the O 1s core level of the O^{2-} lattice peak (0) at time zero (top). The temporal binning is 250 fs.

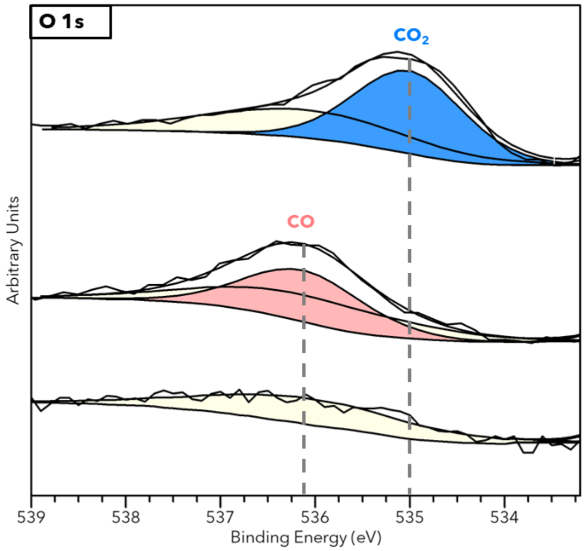

Fig. S3: XP spectra of O 1s core level of the cleaned rutile surface, after CO and after CO_2 adsorption at 98 K. Spectra are recorded with a lab-based Al Kα source ($h\nu_{\mathrm{Mono}}$ = 1486.6 eV, E_p=40 eV) under normal emission. Adsorbed CO (red) shows a component at 536.1 eV and CO_2 (blue) at 535.0 eV on top of the O 1s satellite peak (yellow).

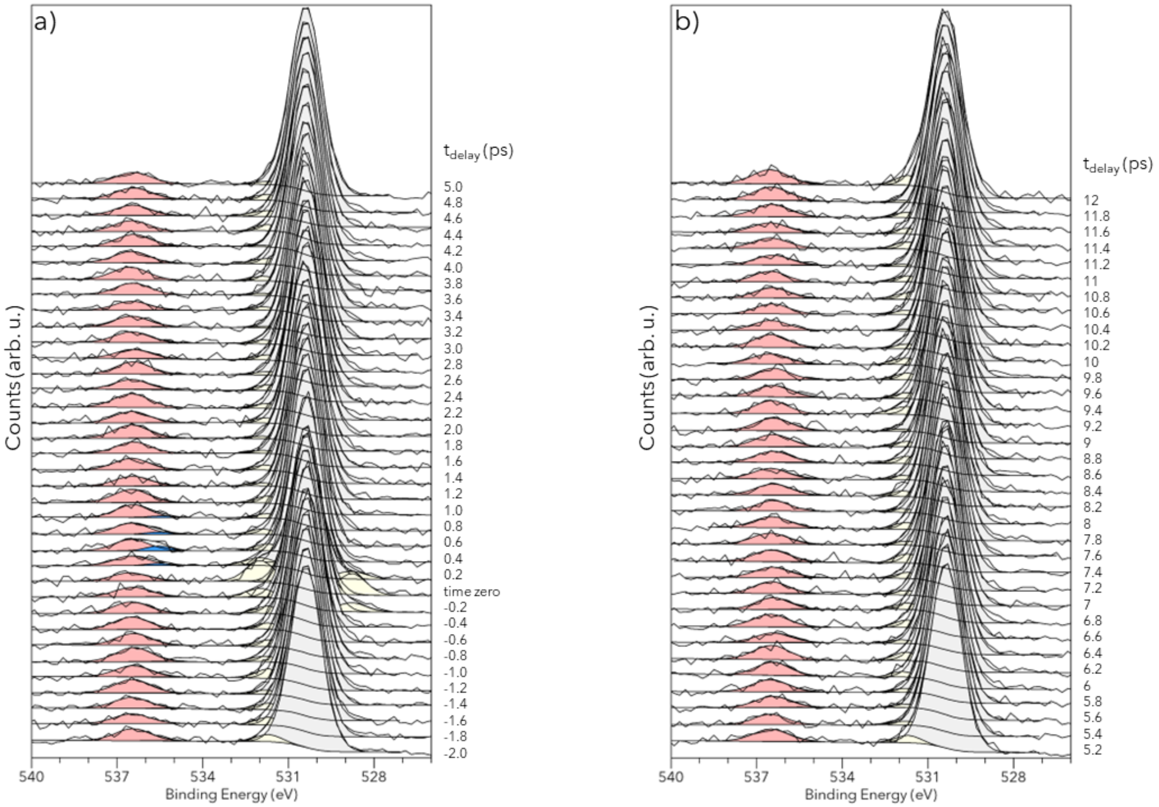

Fig. S4: Time resolved XP spectra of O 1s core level during photooxidation of CO to CO_2 on rutile $TiO_2(110)$ at 80 K binned in 200 fs intervals at the marked time a) from -2 ps to 5 ps and b) from 5.2 to 12 ps. The spectra show no indication of any intermediate species during the CO oxidation with a lifetime longer than 200 fs. Due to the 200 fs binning the sideband are visible in the spectra from -0.2 to 0.2 ps.

System	E_{ads} (eV)
A) CO adsorption	-0.525
B) O_2 adsorption (perpendicular)	-0.132
C) O_2 adsorption (parallel)	-0.105
D) coadsorption CO and O_2 (perpendicular)	-0.667
E) coadsorption CO and O_2 (parallel)	-0.505

Table S1: Energetic values obtained by DFT periodic calculations for the adsorption energies in eV per adsorbed molecule in the systems depicted in the FigureS7 below.

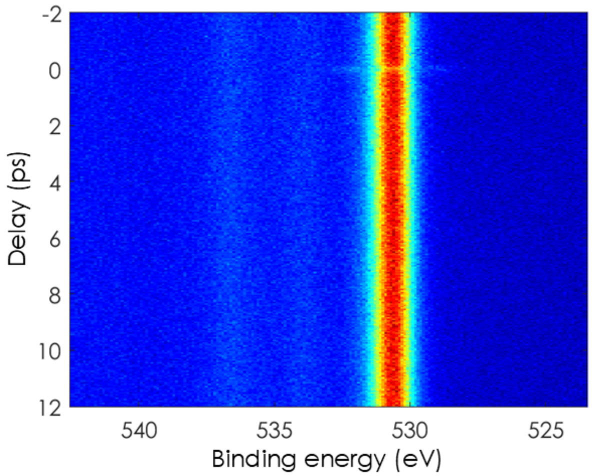

Fig. S5: The tr-XP map of the energy region of O 1s core level taken at FLASH. The color scale indicates the intensity of the XP spectra, with the TiO_2 O 1s lattice peak at 530.4 eV. At time zero the intensity of the lattice peak decreases and sidebands appear 1.6 eV ahigher and lower binding energy. The binning size is 0.05 ps for the delay and 0.1 eV for the binding energy.

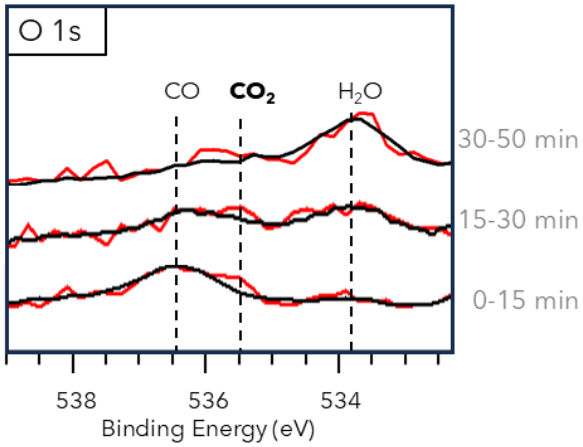

Fig. S6: Average (black) and time-resolved from 0.25 to 0.75 ps after initiation (red) O 1s spectra during CO oxidation binned in 0-15 min, 15-30 min, and 30-50 min after flash-annealing. The CO_2 signal from 0.25 to 0.75 ps is visible in two datasets with different water coverages assigned to the ultrafast oxidation of CO.

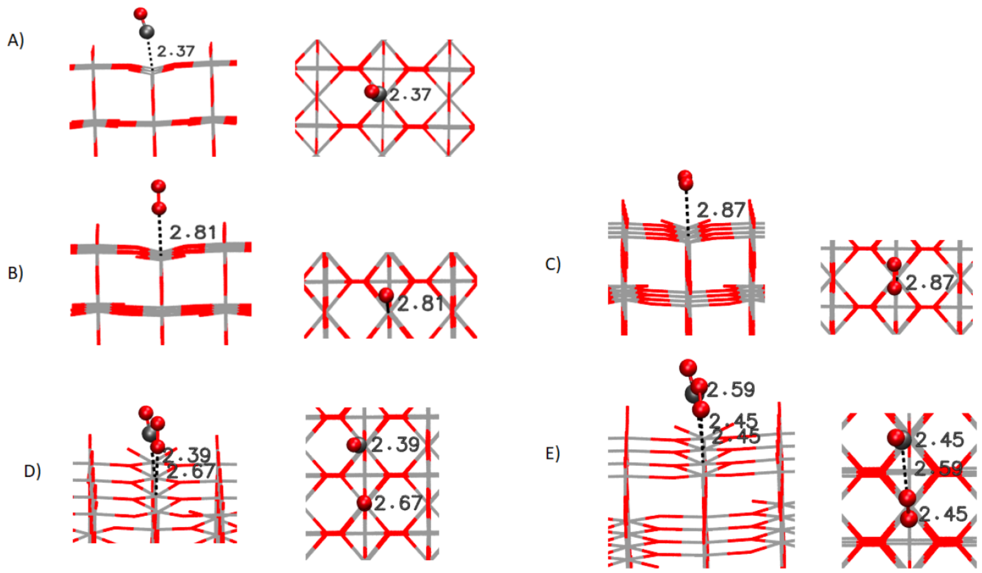

Fig. S7: Snapshots of the optimized geometry obtained (left side view, right top view, respectively) corresponding with: A) CO adsorption; B) O_2 adsorption (perpendicular configuration); C) O_2 adsorption (parallel configuration); D) coadsorption of CO and O_2 (perpendicular configuration); E) coadsorption of CO and O_2 (parallel configuration). Bond lengths are given in Angstrom(Å). The optimized geometries and energetics for CO and O_2 underline the preference of both molecules to interact with the pentacoordinated Ti (Ti_{5c}) atoms, in agreement with previous reports. [1,2] The chemistry of CO is ruled by the intrinsic properties of the CO molecule with the C atom presenting a negative electronic partial charge based on the Lewis structure, explaining the C atom ending orientation in the interaction with Ti atoms. Two different configurations were considered for the adsorption case of O_2 on TiO_2 rutile (110) surface. The first one with the perpendicular O_2 molecule interacting with an interatomic distance between the Ti_{5c} and the nearest molecular oxygen of 2.81 Å and a binding energy value of -0.132 eV. The second one with the parallel O_2 molecule interacting with an interatomic distance between the Ti_{5c} and the nearest molecular Oxygen of 2.87 Å and a binding energy value of -0.105 eV.

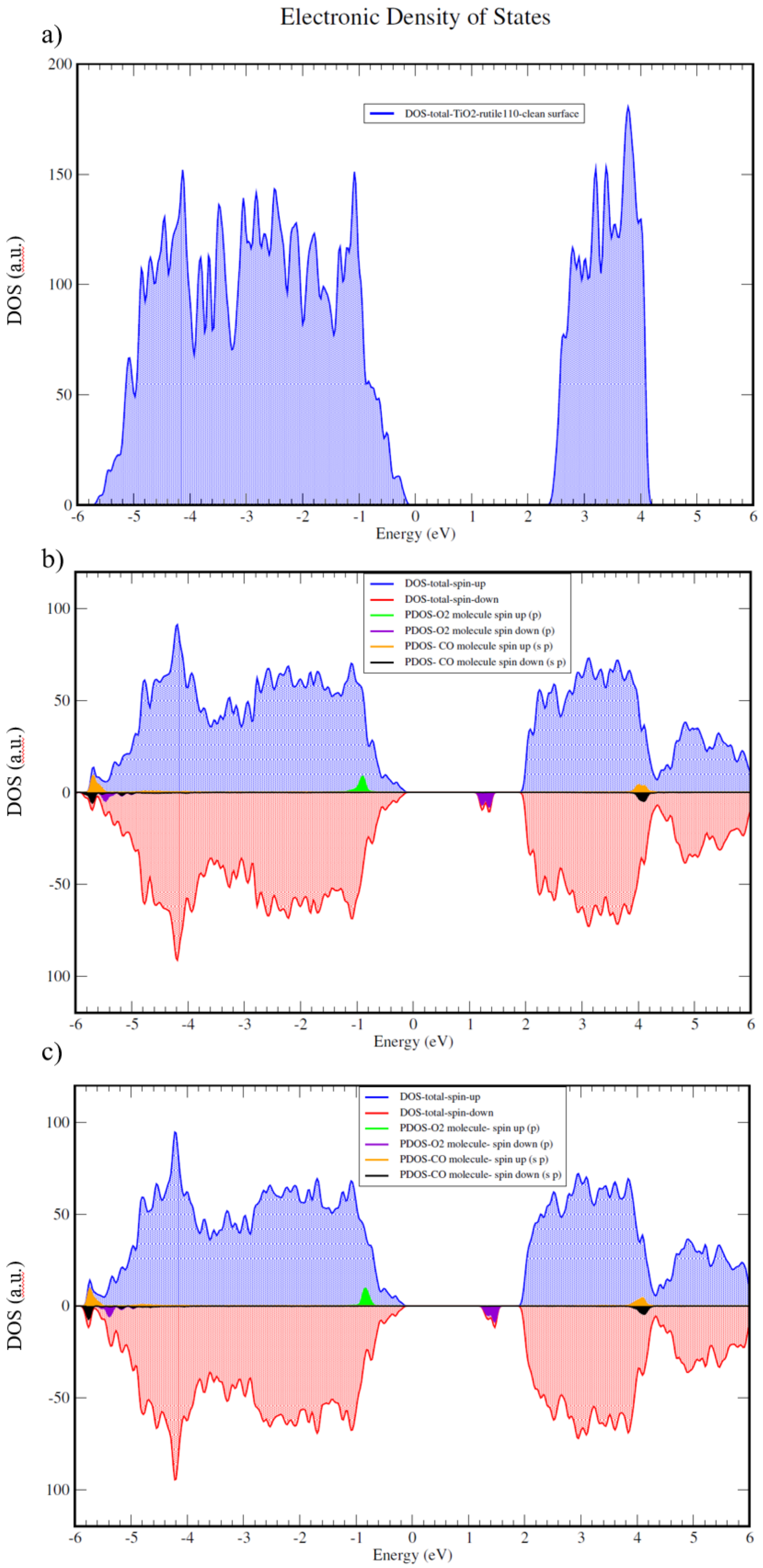
Electronic Density of States
a)
DOS-total-TiO2-rutile110-clean surface
DOS (a.u.)
Energy (eV)
b)
DOS-total-spin-up
DOS-total-spin-down
PDOS-O2 molecule spin up (p)
PDOS-O2 molecule spin down (p)
PDOS- CO molecule spin up (s p)
PDOS- CO molecule spin down (s p)
DOS (a.u.)
Energy (eV)
c)
DOS-total-spin-up
DOS-total-spin-down
PDOS-O2 molecule- spin up (p)
PDOS-O2 molecule- spin down (p)
PDOS-CO molecule- spin up (s p)
PDOS-CO molecule- spin down (s p)
DOS (a.u.)
Energy (eV)

Fig. S8: previous page: Density of states (DOS) of a) the pristine TiO_2 rutile 110 surface, b) the coadsorption of CO and O_2 in parallel configuration and c) the coadsorption of CO and O_2 in perpendicular configuration, using PBE functional at the DFT level of theory with the semi-empirical nonlocal external potentials. Additionally, the projections of the DOS on the adsorbate orbitals are displayed.

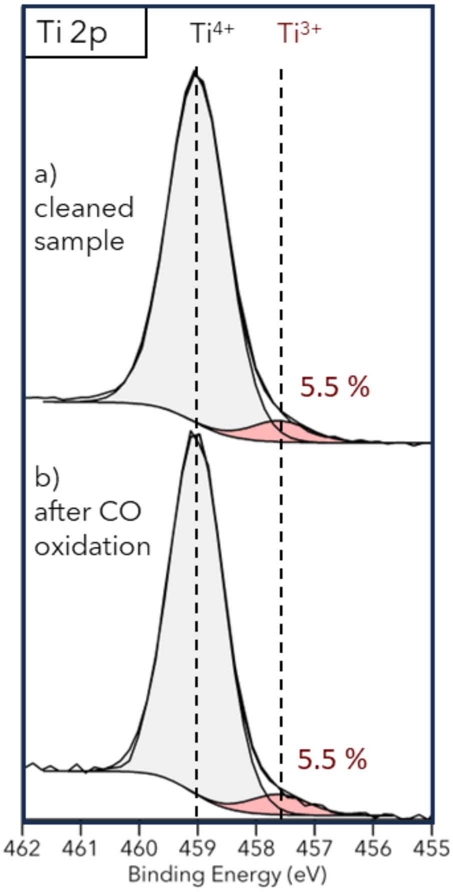

Fig. S9: Ti 2p spectrum a) after cleaning in oxygen at 650 °C before CO oxidation and b) after several cycles of CO oxidation at 80 K. The defect concentration (determined by the Ti^{3+} component) does not change during the CO oxidation

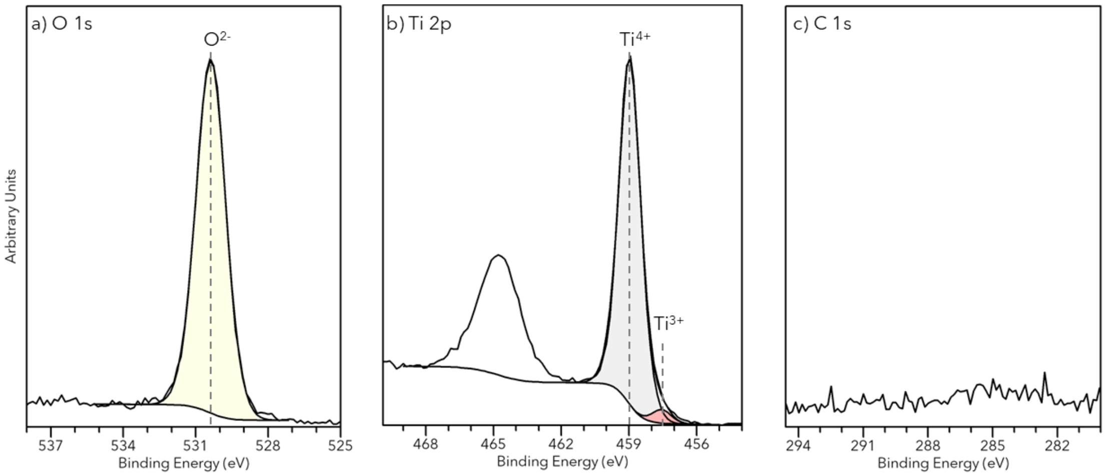

Fig. S10: XP Spectra ($h\nu_{FEL}$ = 643 eV, T = 293 K) of a) O 1s, b) Ti 2p and c) C 1s of the cleaned TiO_2(110) surface. The O 1s spectra show the O^{2-} lattice peak. The Ti 2p spectrum consists of the Ti^{4+} $2p_{3/2}$ at 459 eV and the broader Ti^{4+} $2p_{1/2}$ peak. The Ti^{4+} $2p_{3/2}$ peak exhibits a slight asymmetry on the lower binding energies, which is assigned to a low amount of Ti^{3+} defects. The C 1s spectrum showed no contaminants on the cleaned surface.

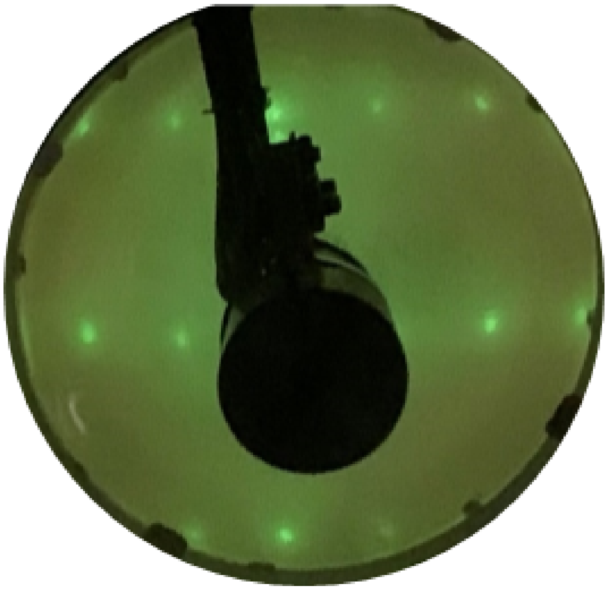

Fig. S11: (1x1) LEED pattern of prepared rutile TiO_2(110) surface.

References

[1] Amy Linsebigler, Guangquan Lu, and John T. Yates. CO chemisorption on TiO_2(110): Oxygen vacancy site influence on CO adsorption. J. Chem. Phys., 103(21):9438–9443, 1995.

[2] Zdenek Dohnálek, Jooho Kim, Oleksandr Bondarchuk, J. Mike White, and Bruce D Kay. Physisorption of N_2, O_2, and CO on fully oxidized TiO_2 (110). J. Phys. Chem. B, 110(12):6229–6235, mar 2006.
