# Supplementary material for: Dynamics of CO photooxidation to CO2 on rutile (110)
Source: Commun Chem. 2026 Mar 10;9:127. doi: 10.1038/s42004-026-01901-2 (PMC13009378; doi:10.1038/s42004-026-01901-2)
Supplement: Supplementary file 3 — Description of Additional Supplementary Files [file 42004_2026_1901_MOESM3_ESM.pdf]

Description of Additional Supplementary Files:

File name: Supplementary Data 1

Description: Here We share the .vms file of the XPS data and all related analysis of these data.
